# Supplementary material for: History-dependent domain and skyrmion formation in 2D van der Waals magnet Fe3GeTe2
Source: Nat Commun. 2022 May 31;13:3035. doi: 10.1038/s41467-022-30740-7 (PMC9156682; doi:10.1038/s41467-022-30740-7)
Supplement: Supplementary file 1 — Supplementary Information [file 41467_2022_30740_MOESM1_ESM.pdf]

Supplementary Information: History-dependent domain and skyrmion formation in 2D van der Waals magnet Fe_3GeTe_2

M. T. Birch[1,8], L. Powalla[2,8], S. Wintz[1], O. Hovorka[3], K. Litzius[1], J. C. Loudon[4], L. A. Turnbull[5], V. Nehruji[3], K. Son[1,6], C. Bubeck[1], T. G. Rauch[7], M. Weigand[7], E. Goering[1], M. Burghard[2], G. Schütz[1]

[1]*Max Planck Institute for Intelligent Systems, 70569 Stuttgart, Germany*

[2]*Max Planck Institute for Solid State Research, 70569 Stuttgart, Germany*

[3]*Faculty of Engineering and Physical Sciences, University of Southampton, Southampton SO17 1BJ, UK*

[4]*Department of Materials Science and Metallurgy, University of Cambridge, Cambridge, CB3 0FS, UK*

[5]*Department of Physics, Durham University, Durham, DH1 3LE, UK*

[6]*Department of Physics Education, Kongju National University, Gongju 32588, South Korea*

[7]*Helmholtz-Zentrum Berlin für Materialien und Energie GmbH, Institut Nanospektroskopie, 12489 Berlin, Germany*

[8]*These authors contributed equally to the work.*

Supplementary Note 1: Bulk sample characterisation and flake sample fabrication

Energy dispersive x-ray spectroscopy (EDX) measurements were performed on the bulk FGT single crystal sample. The spatially resolved results for each element are shown in Fig. S1, demonstrating good compositional homogeneity, and yielding an elemental composition of $Fe_{2.95}GeTe_{1.70}$. We performed measurements of the magnetisation as a function of temperature with the magnetic field applied along both the out-of-plane ($H \parallel c$) and in-plane ($H \perp c$) directions of the FGT crystal lattice, as shown in Fig. S2. We performed magnetisation versus temperature measurements following two measurement procedures: measuring while field cooling (FC) from 300 K to 5 K at applied fields of 30 Oe and 300 Oe; and measuring while field-warming from 5 K to 300 K, again at 30 Oe and 300 Oe, after initialising the sample by zero field cooling (ZFC) to 5K. The results are shown in Fig. S2a-d.

Measurements of magnetisation as a function of the applied field were performed for both field-sample configurations, with a selection of the resulting data shown in Fig. S2e-h. Immediately, one can see that the strong uniaxial crystalline anisotropy results in the c direction being the easy axis. The uniaxial anisotropy can be estimated by considering the magnetic work done, W, to saturate the system at the saturation magnetisation, M_s, along each crystal orientation $\hat{H}$,

$$W_{\hat{H}} = \mu_0 \int_0^{M_\mathrm{s}} H dM. \quad (1)$$

Thus, by integrating over the magnetisation versus field curves, the anisotropy can be calculated, $K = W_{H\parallel c} - W_{H\perp c}$. The anisotropy and saturation magnetisation, M_s, extracted as a function of temperature are shown in Fig. S2i and j respectively. The result demonstrates the large, roughly

linear, increase of K with decreasing temperature. In the mean-field simulations, we utilised a similar scaling of K with temperature to recover the temperature dependent size of the observed magnetic domains, which at low enough temperatures leads to uniform switching of the simulated sample, as was observed in the real-space measurements of the FGT flake.

Having characterised the bulk material, we proceeded to fabricate an FGT flake sample using the mechanical exfoliation technique. The optical microscope images in Fig. S3a and b show the chosen exfoliated FGT and hBN flakes on PDMS stamps before the transfer. Fig. S3c the completed heterostructure after stamping first the FGT and then the hBN flake on to the Si_3N_4 membrane.

Supplementary Note 2: Flake thickness determination

We utilised a combination of AFM and x-ray absorption analysis to estimate the thickness of each region of the FGT flake. Fig S4a shows an AFM image of a corner of the investigated FGT flake. Cuts across the steps of three thickness regions are labelled S1-3. The profile of each step is shown in Fig. S4b, revealing thicknesses of approximately 60, 30 and 15 nm, as well as a measure of the hBN capping flake thickness of roughly 15 nm. A STXM image of the flake is shown in Fig. S4c, with a number of regions numbered 1 to 5 in order of decreasing thickness, including a 6th point passing through only the Si_3N_4 membrane, which acts as a measure of the incident beam intensity, or I_0 signal. The ROI investigated in the main text is indicated, and the thicknesses of the regions measured in the AFM data are labelled. Absorption spectra measured locally in each

thickness region, across the L_3 edge, are plotted in Fig. S4d. We extracted the final data point in these spectra, measured away from the resonant edge at 715 eV, and divided them by the I_0 value to determine the x-ray transmission, T, of each thickness region. These transmission values are plotted on a logarithmic scale as a function of sample thickness in Fig. S4e, where the solid line is a linear fit to Beer's law. Using this calibration, we determined that the three thicknesses featured in the ROI were approximately 60, 50 and 35 nm respectively.

Supplementary Note 3: X-ray absorption spectra results and oxide thickness

To investigate the oxidisation of our samples, x-ray absorption spectra of both the bulk and flake FGT samples were measured. In Fig. S5a we show the Fe L_3 and L_2 edge absorption of the bulk FGT sample measured using the surface-sensitive total electron yield (TEY) method (see methods). The spectra were processed by first rebinning the measured data to a 0.1 eV energy resolution. Then, the data was divided by the I_0 (the measured intensity of the synchrotron beam at that energy) measurement to isolate the signal from the sample. Next, a background was subtracted by performing a linear fit to the first 5 eV of the data set. Finally, the spectra was edge-normalised, as is standard practice, by dividing to set the after-edge value to unity.

Spectra were measured for both an initially uncleaved surface, and for a surface cleaved under vacuum conditions. The large secondary peak in the uncleaved spectrum reveals the change in the valence state of the Fe^{3+} to Fe^{2+} due to reaction with oxygen under ambient conditions. The uncleaved surface showed no XMCD signal at 80 K, indicating oxidised FGT is non-magnetic.

We also measured absorption spectra at the O K edge for both the cleaved and uncleaved samples, as shown in Fig. S5b. The lack of absorption at the O K edge demonstrates that by cleaving the sample in vacuum, almost all oxidised FGT material can be removed. Comparison of the results in Fig. S5a and b, confirms that the large secondary absorption peak at the Fe L_3 edge in the FGT is due to oxidisation of the Fe.

In the cleaved sample spectrum, a strong XMCD signal was observed by measuring spectra at oppositely applied magnetic fields, as shown in Fig. 5c. Interestingly, there are still two clear peaks in the L_3 absorption edge, which we attribute to the two Fe ion species present in the FGT structure, commonly referred to as the Fe I and Fe II sites[1]. Fig. 5c also shows the ad hoc step function utilised to subtract the background from excitations into continuum states, where the height of the steps at the L_3 and L_2 edges was set at a ratio of 2:1, according to the occupation of the $2p_{3/2}$ and $2p_{1/2}$ core states. Having subtracting this non-resonant background, the resonant contribution of the absorption spectrum, and its integrated value, is plotted in Fig. 5d. Finally, the integrated XMCD spectrum is plotted in Fig. 5e. Using sum rules, the spin and orbital contributions of the magnetic moment, m_s and m_o, can be determined from the integrated resonant absorption and XMCD spectra[2]. We recovered values of 1.52 μ_B and 0.05 μ_B for m_s and m_o respectively, comparable to other theoretically and experimentally determined values found in the literature[2,3]. These corresponds to a value of approximately 375 kA/m in the bulk sample, which is comparable to that measured in the magnetometry data.

Spectra from $\sim$10 and 60 nm regions of the flake sample are shown in Fig. 6a and b, mea-

sured at 100 K. The lack of XMCD effect in the 10 nm region illustrates the absence of magnetic order in this thickness, while the large secondary peak indicates significant oxidisation of the sample, as was seen in the uncleaved bulk spectrum. On the other hand, the spectra measured in the 60 nm region shows a strong XMCD signal, indicating magnetic order, and a significantly reduced second Fe absorption peak, suggesting less overall oxidisation of the sample. Given that all thickness regions of 15 nm and below were observed to be non-magnetic, and since the oxidisation of the sample is likely to be uniform across the entire surface of the flake, we can estimate that roughly 7–10 nm of both the top and bottom surfaces of the flake sample were oxidised before being capped by the hBN layer.

Once again, we performed XMCD sum rules analysis on the 60 nm flake spectrum, using the integrated values show in Fig. S6c and d. We recovered values of 1.02 μ_{B} and 0.08 μ_{B} for m_{s} and m_{o} respectively. Using the values of m_{s} acquired from the bulk spectrum, 1.52 μ_{B}, we can estimate that 40 nm of the the 60 nm thickness region remains magnetic, implying that there is a 10 nm oxide layer on the top and bottom surface of the flake, agreeing with the previous estimate. This would leave the magnetic thickness of the areas in the region of interested featured in the main text as 15, 30 and 40 nm.

To directly investigate the oxidised FGT layer thickness, we performed standard transmission electron microscopy (TEM) to examine the thickness of the oxide layer in the FGT flakes fabricated by our exfoliation method in ambient conditions. Due to the challenge of acquiring a TEM lamella using focused ion beam from a flake sample stamped on a thin Si_3N_4 membrane, we prepared

an additional flake sample, capped by hBN, prepared on a SiO_2 substrate. Besides the change of substrate, the fabrication method was identical, with the flake being exposed to ambient conditions for about 30 mins. Focused ion beam was utilised to prepare a lamella of a cross section through this flake stack. Layers of carbon and Pt were deposited before the milling and lift out procedure to protect the underling structure of the sample from ion damage. The resulting TEM images are shown in Fig. S7a and b, where the square root of the intensities has been shown to improve the contrast of each material layer. By the lightening of the contrast, indicating lower average atomic number, and the breakdown of the clean vdW layers, within the FGT section, the oxidised FGT layer thickness can be estimated to be about 7 nm at the top and bottom of the flake. It is clear that the oxidised FGT does not form a clean interface with the pristine FGT. This can be expected, since one might assume that the penetration of the oxygen into the bulk of the FGT would be some form of exponentially decaying process.

Supplementary Note 4: Extended STXM imaging data

We performed STXM imaging following the zero field-cooling (ZFC) procedure to map out the phase diagrams presented in the main text. The results when ZFC to 150 and 125 K, and the subsequent decrease in field, are shown in Fig. S8a and b respectively. One can see that the stripe domains were frozen-in as the sample was cooled. After annihilating the stripes by saturating the magnetisation at these temperatures below 150 K, further field sweeps resulted in the entire sample switching simultaneously, as in the field-sweep protocol. We found it was possible to freeze-in domains at 0 mT in this way down to 30 K – the lowest temperature achievable in our

STXM instrument. Beyond those presented in the main text, we acquired STXM images at further temperatures following the field sweep procedure, as shown in Fig. S9. In the case of the data at 186 K, only the 60 nm thickness region showed visible magnetic domain contrast, and so we focused our imaging measurements solely on this region.

In the main text, we present the majority of the STXM images after normalising the contrast within each thickness region. This allows the magnetic contrast to be discerned more clearly, by eliminating the structural contrast due to the different thickness layers. The contrast normalisation was performed by isolating each thickness region of the flake using a mask, as shown in Fig. S10a-c. By summing these masked images together, we achieve the result shown in Fig. S10d, where the signal seen within each thickness region is primarily out-of-plane magnetic contrast.

Supplementary Note 5: Extended LTEM data

We performed Lorentz transmission electron microscopy (LTEM) on a separate FGT flake sample to investigate whether the magnetic textures were Bloch or Néel type. This FGT sample was prepared in a similar manner to those used for x-ray imaging, but the hBN capping layer was not used, since it introduced additional structural contrast to the images. The uncapped FGT sample was kept in an Ar atmosphere between preparation and the LTEM measurement to reduce its exposure to ambient conditions. The inset of Fig. S11a shows an optical micrograph of the FGT flake with the area imaged in the electron microscope outlined in red. The thicknesses exhibited by this FGT flake are comparable to the sample measured with STXM, showing regions between

15-70 nm thickness. The LTEM image shown in Fig. S11a shows lines of contrast indicating the domain walls associated with magnetic stripe domains.

Two methods were used to distinguish Bloch and Néel domain walls. The first made use of the fact that, unlike Bloch walls, Néel walls produce no contrast in an image when the electron beam is at normal incidence to the sample (0°). For this sample, the structural diffraction contrast close to 0° was too strong to discern any magnetic features as the specimen was oriented close to the [001] zone axis of the crystal. Nevertheless, a comparison between Fig. S11b, acquired with the specimen tilted to 36°, and Fig. S11c, acquired at 10°, shows a dramatic reduction in the magnetic contrast, suggesting the walls are Néel type.

The second method was to compare experimental images with simulated LTEM images. A field of 135 mT was applied to saturate the sample and then reduced to zero whilst the temperature was maintained at 92 K. This created larger domains at zero field so that individual domain walls could be distinguished clearly as shown in Fig. S12a. Simulated images for the same conditions are shown in Fig. S12b and it can be seen that Néel walls produce a single line which can be black or white, depending on the orientation of the wall. Bloch walls, on the other hand, produce a double black-white line for each wall. Since the experimental image in Fig. S12a shows single black or white lines, we conclude that the domain walls are Néel type.

Supplementary Note 6: Determining domain sizes

In the main text, we presented data showing the average stripe domain size as a function of temperature when following the field sweep procedure. For each image in the datasets, we manually acquired five line scans through the data, taking care to choose lines which bisected the domains at a right angle. Example line scans for different temperatures are plotted in Fig. S13a-c, together with the original images where the location of the line is indicated. We then determined the mid point at which the contrast changes from light to dark, as indicated by the yellow and purple coloured regions on each plot. The results were then averaged for each image, resulting in an average domain size from a sample size of approximately 15-20 domain measurements per thickness at each field point. The diameters of the skyrmions plotted in the inset Fig. 4a of the main text were acquired in a similar manner. The determined domain size for each image following the field-sweep procedure is plotted in Fig. S14a-f. The domain wall width, w, were obtained by fitting linescans bisecting stripe domains at 90 degrees to the domain wall profile function $m_z = \tanh(\pi(x - x_0)/w) + c$, where m_z is the observed magnetic contrast, and c is the non-XMCD signal background count rate.

Supplementary Note 7: Field cooled skyrmion fitting

To extract the number and size of the skyrmions from the images obtained after FC, fitting was performed using the OpenCV Python Library[4]. Fig. S15a shows the contrast-normalised image after FC at 15 mT from above T_C to 125 K. To begin, thickness regions were isolated in the raw data using an intensity threshold, as shown for the 60 nm region in S15b. Next, the edges of the

skyrmions were located using a Canny edge detection algorithm, resulting in the output shown in Fig. S15c. These output fits were refined by noise filtering and manually excluding ill-fitting contours. The contour functionality of the OpenCV library was used to fit contours to the bubble edges, and extract their areas – the results for each thickness region are shown in S15d and e. We excluded all skyrmions that were not wholly within the image, and also excluded skyrmions within the narrow portion of the 50 nm thickness region, due to the difficulty of isolating magnetic objects in this area. The final determined contours of the skyrmions are overlaid on the original contrast normalised STXM image in Fig. S15f. The data utilised to plot the size and number of skyrmions after field cooling at a range of fields, in Fig. 5k of the main text, are shown in Fig. S16c.

Supplementary Note 8: Mean-field vs micromagnetic parameters

As discussed in the main text, the mean-field parameter choices of $J_{\mathrm{E}} = 0.7\lambda$, $J_{\mathrm{D}} = 0.1\lambda$ and $J_{\mathrm{K}} = 2.5\lambda$ in Eq. (1) allowed our simulations to qualitatively reproduce the main experimental observations (Fig. 6 in the main text). The scale factor $\lambda = 10^{-20}/\sqrt{3}$ J determines the absolute energy scale. Since this factor multiplies all energy terms in Eq. (1) equally, it does not influence the physical behaviour and can be removed from consideration by normalising Eq. (1) as $\mathcal{H}_{\mathrm{MF}}/\lambda$.

This allows the model parameters, J_{E}, J_{D} and J_{K}, to be related to equivalent micromagnetic parameters. In the temperature limit $T \to 0$, the spin-lattice based mean-field energy $\mathcal{H}_{\mathrm{MF}}$ defined by Eq. (1) can be seen as a discretisation of the standard micromagnetic energy functional. Since we assume the spins in the mean-field model are populated on a 2D hexagonal lattice, the rela-

tion between the mean-model parameters J_{E}, J_{D}, J_{K}, given in energy units (Joule J), and their micromagnetic equivalents A (J/m), D (J/m^2), K (J/m^3) are given by the standard expressions[5],

$$A = \frac{\sqrt{3}J_{\mathrm{E}}m^2(T)}{2a}, \qquad D = \frac{\sqrt{3}J_{\mathrm{D}}m^2(T)}{a^2}, \qquad K = \frac{2J_{\mathrm{K}}}{\sqrt{3}a^3}, \tag{2}$$

where a is the discretisation constant and $m(T)$ average magnetic moment of spins. We will take a to be equal to the Bloch parameter $l_{\mathrm{w}} = \sqrt{A/K}$, which describes the smallest characteristic domain wall size in the material. It assumes small values for hard magnetic materials (e.g. $l_{\mathrm{w}} = 1.3$ nm for bulk $Nd_2Fe_{14}B$) and large values for soft magnetic materials (e.g. $l_{\mathrm{w}} = 18$ nm for bulk Fe)[6]. Fe_3GeTe_2 intuitively falls between these limits and we will take $a = 5$ nm as a reasonable estimate (similar to bulk Co).

The micromagnetic DMI energy constant was estimated in the main text based on experiments to be $D = 0.12 \times 10^{-3}$ J/m^2. Therefore, by using the first two relations in Eq. (2) we obtain $A = (J_{\mathrm{E}}/J_{\mathrm{D}})aD/2 = (7/2)aD \approx 2.1$ pJ/m, where $J_{\mathrm{E}}/J_{\mathrm{D}} = 7$ based on the values given above. Similarly, using the last two relations in Eq. (2) we obtain $K = (2/3)(J_{\mathrm{K}}/J_{\mathrm{D}})D/a \approx 0.4$ MJ/m^3, where $J_{\mathrm{K}}/J_{\mathrm{D}} = 25$. It's crucial to note that these values are the zero temperatures values of the mean-field model, where the simulations demonstrate uniform switching of the magnetisation. Therefore, it is expected that they differ from the parameters utilised in the micromagnetic simulations, where we are simulating skyrmions at a higher temperature, and therefore require a smaller value of the anisotropy.

Supplementary Note 9: Temperature dependence of anisotropy in the mean-field model

To describe the temperature dependence of anisotropy in the mean-field model we first considered the classical Callen-Callen theory[7], based on which we represented uniaxial anisotropy constant associated with individual magnetic moments as:

$$J_{\mathrm{K}}(T) = J_{\mathrm{K}}(0)\left(\frac{m(T)}{m(0)}\right)^{n} = J_{\mathrm{K}}(0)m^{n}(T) \tag{3}$$

where $m = |\mathbf{m}|$ and $m(0) = 1$. In simulations we set $J_{\mathrm{K}}(0) = 2.5$ (consistent with the micromagnetic value $K = 0.4$ MJ/m^3 as discussed in Supplementary Note 8) and the exponent $n = 2$.

While Eq. (3) reproduced very well the observed experimental trend at higher temperatures, including the formation of skyrmion lattices close to T_c and the mixed skyrmion-stripe domains at intermediate temperatures, the rate of anisotropy increase with decreasing temperatures as given by the current form of Eq. (3) was insufficient to lead to switching between uniform magnetisation states, as was observed in experiments at low temperatures, and as shown by the results in Supplementary Fig. S17. Inspired by the strong temperature dependence of the anisotropy seen in the magnetometry measurements, we therefore modified Eq. (3) by introducing a temperature dependent prefactor to scale the uniaxial anisotropy as:

$$J_{\mathrm{K}}(\beta) = \begin{cases} (c_1(\beta - \beta_1) + J_{\mathrm{K}}(0))m^2(\beta) & \text{if} \quad \beta \geq \beta_1 \\ J_{\mathrm{K}}(0)m^2(\beta) & \text{if} \quad \beta < \beta_1 \end{cases} \tag{4}$$

Thus, Eq. (4) incorporates a piece-wise continuous linear trend in the multiplicative prefactor to enhance the rate of increase of anisotropy at low temperatures below threshold temperature β_1, where $\beta = 1/k_{\mathrm{B}}T$, as demonstrated in Fig. S18. Although the form of Eq. (4) represents only

a simple linear extension of Eq. (3) and is chosen purely on empirical basis, it reproduced the experimentally observed features very well as can be seen in Fig. 6 in the main text. To obtain Fig. 6, the free parameters in Eq. (3) were identified by a trial-and-error process to be equal to $\beta_1 = 1.67\beta_{\rm C}$ and $c_1 = 1.67 J_{\rm K}(0)\beta_{\rm C}$ with $J_{\rm K}(0) = 2.5$.

Finally, it turned out that to describe the observed experimental data trends it was also necessary to enhance the temperature-dependence of exchange in Eq. (1) as:

$$J_{\rm E}(\beta) = \begin{cases} c_2(\beta - \beta_1) + J_{\rm E}(0) & \text{if} \quad \beta \geq \beta_1 \\ J_{\rm E}(0) & \text{if} \quad \beta < \beta_1 \end{cases} \tag{5}$$

To obtain Fig. 6, the free parameters required in Eq. (5) were again identified by a trial-and-error process to be equal to $c_2 = 4.167 J_{\rm E}(0)\beta_{\rm C}$ with $J_{\rm E}(0) = 0.7$.

1. Deiseroth, H.-J., *et al.* Fe_3GeTe_2 and Ni_3GeTe_2 – Two New Layered Transition-Metal Compounds: Crystal Structures, HRTEM Investigations, and Magnetic and Electrical Properties. *Eur. J. Inorg. Chem.* **2006,** 1561 (2006).

2. Zhu, J.-X.,*et al.* Electronic correlation and magnetism in the ferromagnetic metal Fe_3GeTe_2. *Physical Review B* **93,** 144404 (2016).

3. Sun, J., Liu, S., Xiu, F. & Liu, W. Bulk-like Magnetic Moment of Epitaxial Two-dimensional Superlattices. *IEEE Transactions on Magnetics*, doi: 10.1109/TMAG.2021.3084747 (2021).

4. Bradski, G. The OpenCV Library. *Dr. Dobb's Journal of Software Tools* (2000).

5. Cortés, D. I. Computational simulations of complex chiral magnetic structures. *PhD dissertation*, University of Southampton (2017).

6. Bertotti, G. Hysteresis in magnetism: for physicists, materials scientists, and engineers. *Gulf Professional Publishing* (1998).

7. Chatterjee, B. K., Ghosh, C. K. & Chattopadhyay, K. K. Temperature dependence of magnetization and anisotropy in uniaxial $NiFe_2O_4$ nanomagnets: deviation from the Callen-Callen power law. *Journal of Applied Physics* **116,** 153904 (2014).

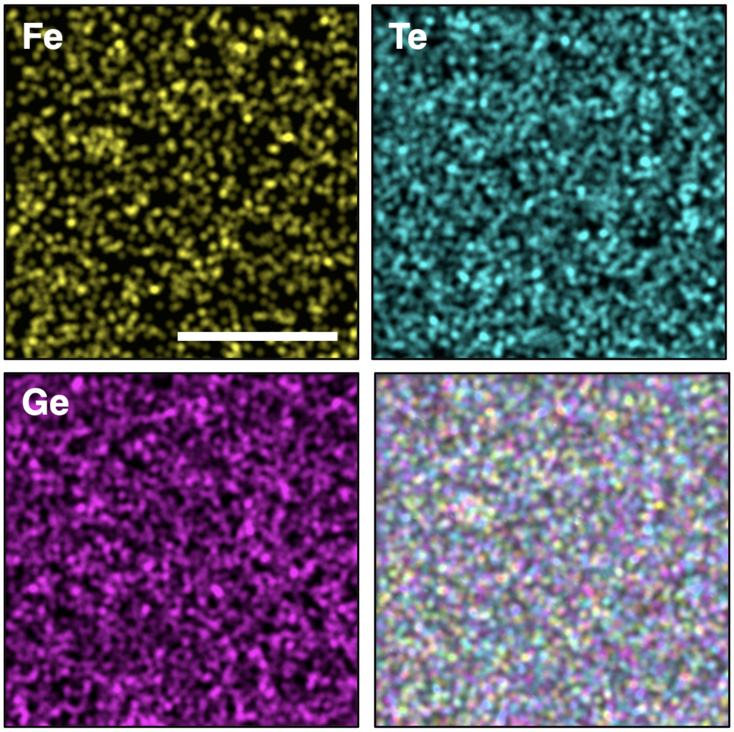

Figure S1 | **Fe_3GeTe_2 sample EDX characterisation.** EDX measurements to determine the Fe, Te and Fe composition of the bulk Fe_3GeTe_2 (FGT) single crystal sample. The Scale bar is 10 μm.

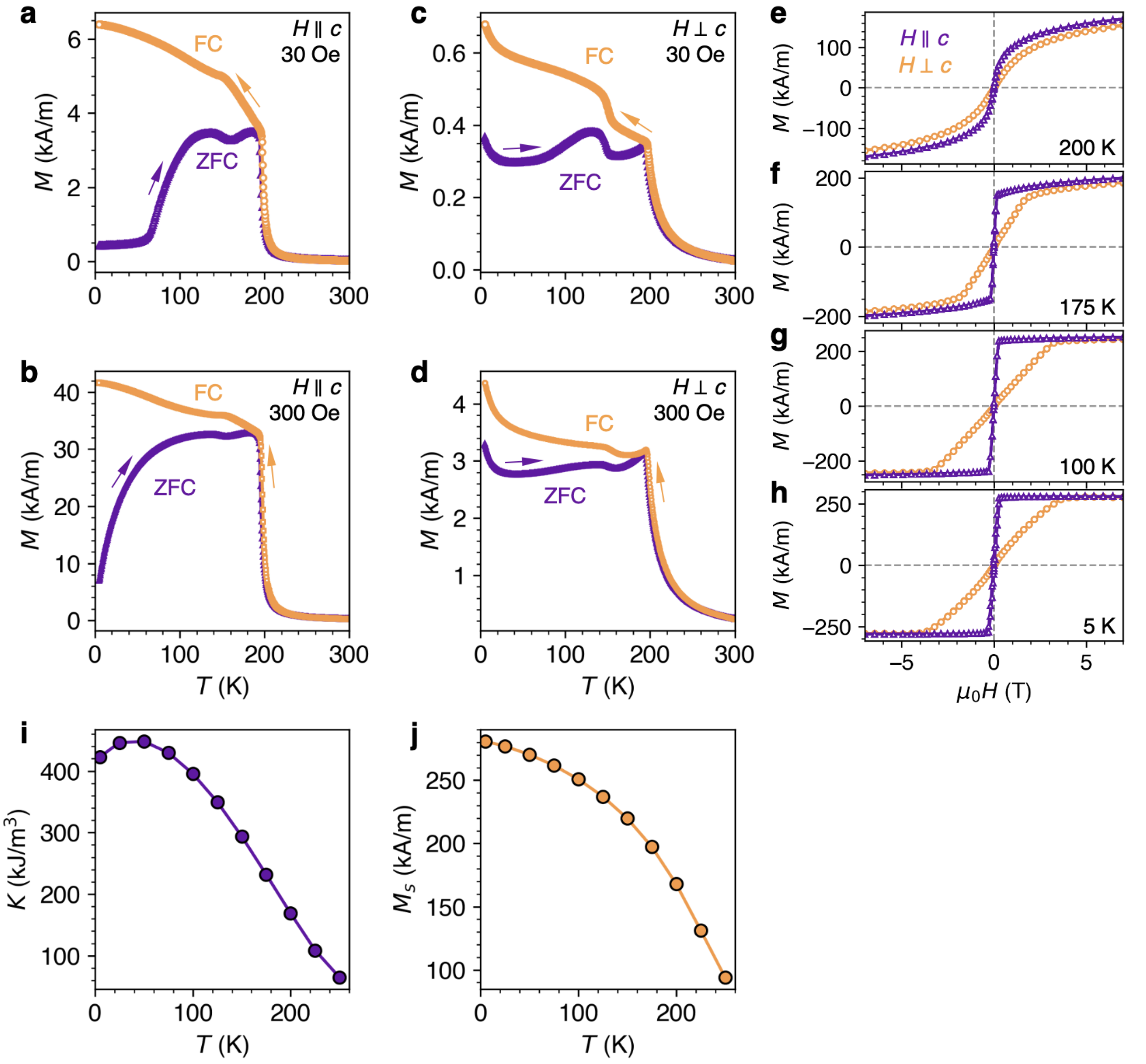

Figure S2 | **Bulk sample magnetometry measurements. a-d**, Magnetisation versus temperature measurements measured with the field aligned parallel, $H \parallel c$ (**a**,**b**), or perpendicular, $H \perp c$ (**c**,**d**), to the c axis of the bulk Fe_3GeTe_2 crystal. Measurements were performed either by field-cooling (FC, orange) from 300 K to 5 K under an applied field, or by initialising the sample by zero field-cooling (ZFC, purple) and subsequently measuring from 5 K to 300 K under an applied field, as indicated by the coloured arrows. Data was measured for applied fields of both 30 Oe (**a**,**c**) and 300 Oe (**b**,**d**). **e-h**, Magnetisation versus field measurements carried out at a selection of temperatures, for both $H \parallel c$ (purple) and $H \perp c$ (orange) field alignments. **i**,**j**, Values of the uniaxial anisotropy, K, and the saturation magnetisation, M_s, extracted from the magnetisation versus field measurements, plotted as a function of temperature, T.

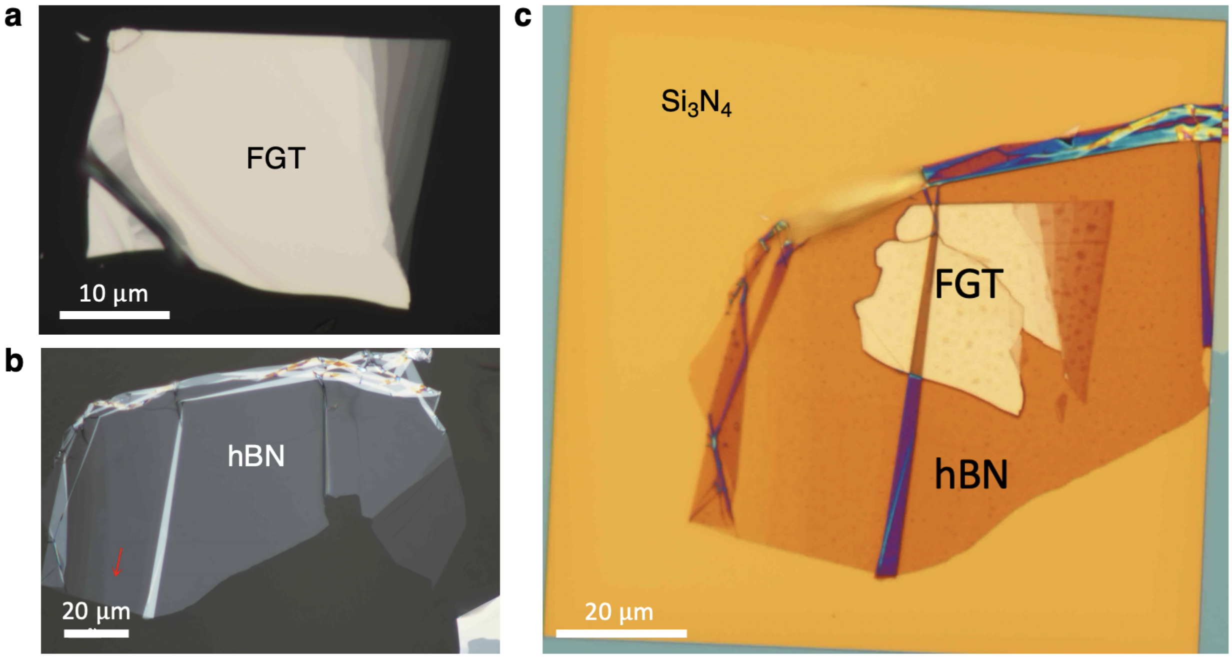

Figure S3 | **Flake sample construction.** **a**, **b**, Optical microscope images of the exfoliated Fe_3GeTe_2 (FGT) and hexaboron nitride (hBN) flakes on PDMS stamps. **c**, Optical microscope image of the completed FGT flake sample, stamped onto the Si_3N_4 membrane and capped with the hBN flake.

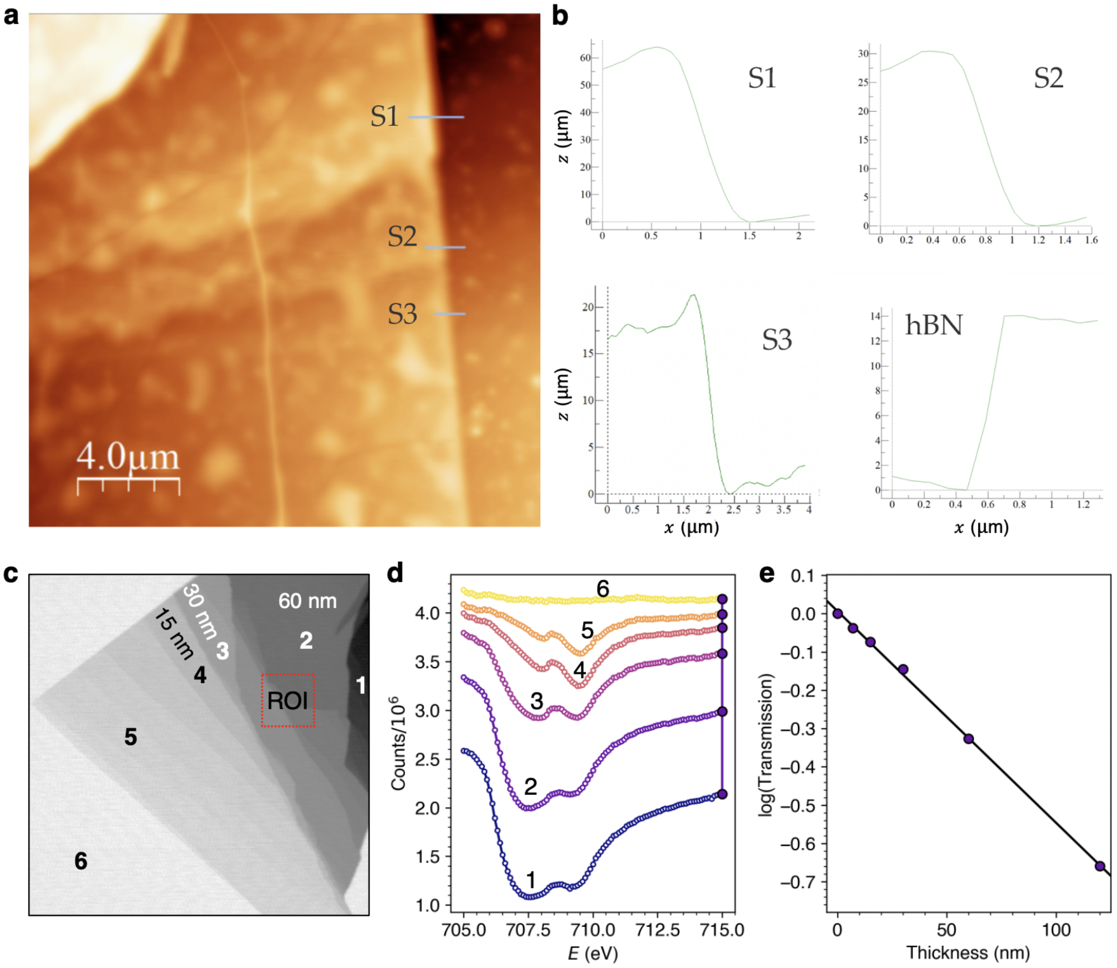

Figure S4 | **Flake thickness determination.** **a**, Atomic force microscopy (AFM) image of a portion of the Fe_3GeTe_2 (FGT) flake sample. **b**, Step height measurements in the sample locations S1, S2, S3 as labelled in **d**, and the thickness of the hexaboron nitride (hBN) layer. **c**, X-ray microscopy image of the FGT flake with the thickness regions measured by the AFM labelled. Further thickness regions are labelled 1 to 6. **d**, X-ray absorption spectra measured in each of the 6 thickness regions labelled in **c** across the Fe L_3 edge. **e**, The transmission of the flake is plotted on a log scale as a function of the determined sample thickness, and fitted with Beer's law, providing a calibration to determine the thickness of all regions.

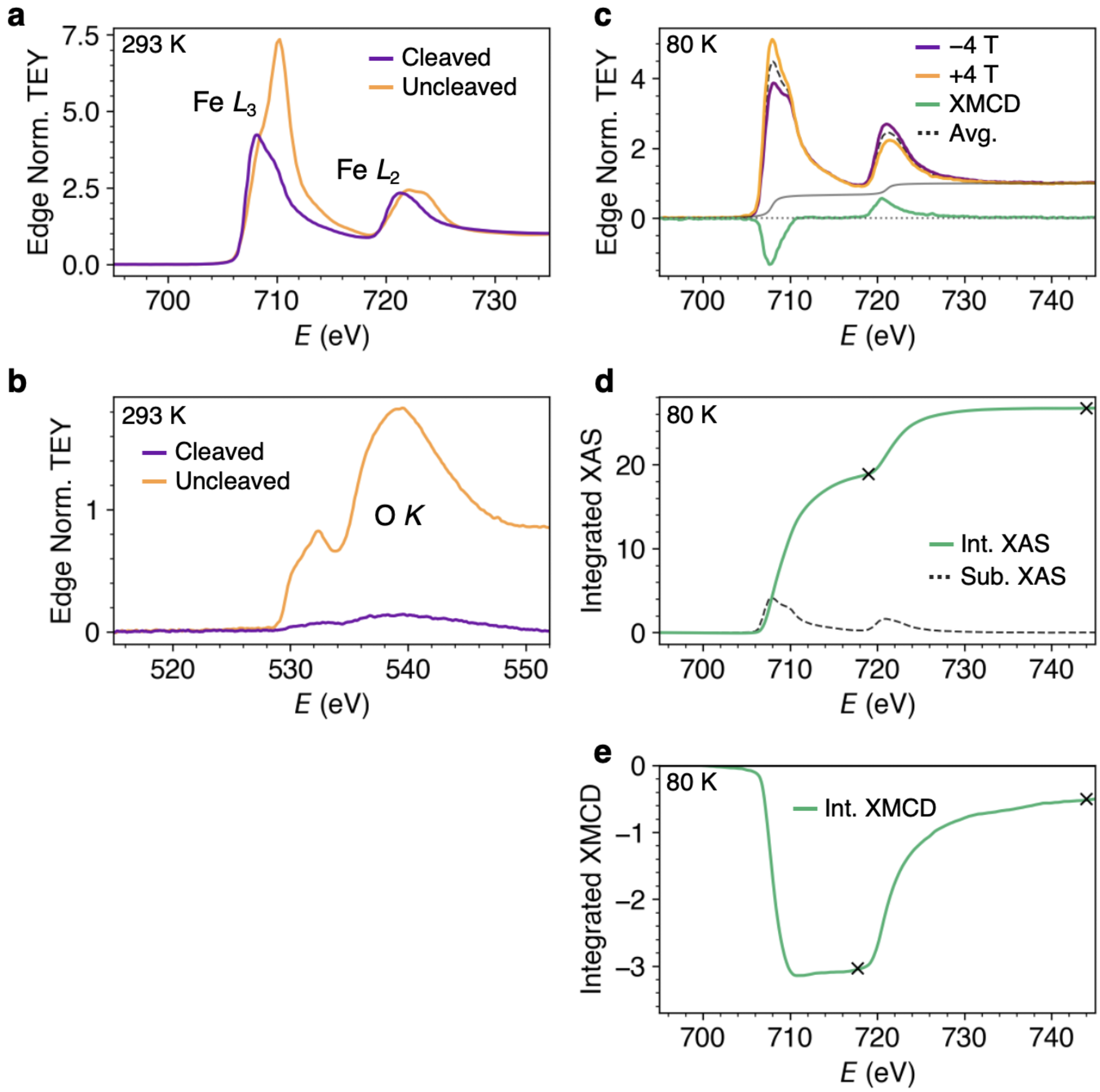

Figure S5 | **Bulk sample x-ray absorption spectra measured with total electron yield.** **a**, X-ray absorption spectra measured at the Fe L_3 and L_3 edges on the uncleaved (orange) and cleaved (purple) Fe_3GeTe_2 (FGT) bulk single crystal at 293 K. **b**, X-ray absorption spectra (XAS) measured at the O K edge on the uncleaved (orange) and cleaved (purple) FGT bulk single crystal at 293 K. **c**, X-ray absorption spectra measured at the Fe L_3 and L_3 edges on the cleaved sample at an applied field of $\pm$ 4 T (orange and purple) at 80 K. Subtraction of the two data sets gives the x-ray magnetic circular dichroism (XMCD) signal (green). **d**, Step-subtracted (black), and integrated (green), x-ray absorption spectrum. **e**, The integrated XMCD signal from **c**. Crosses indicate points in the integrated spectra utilised in the sum rules analysis.

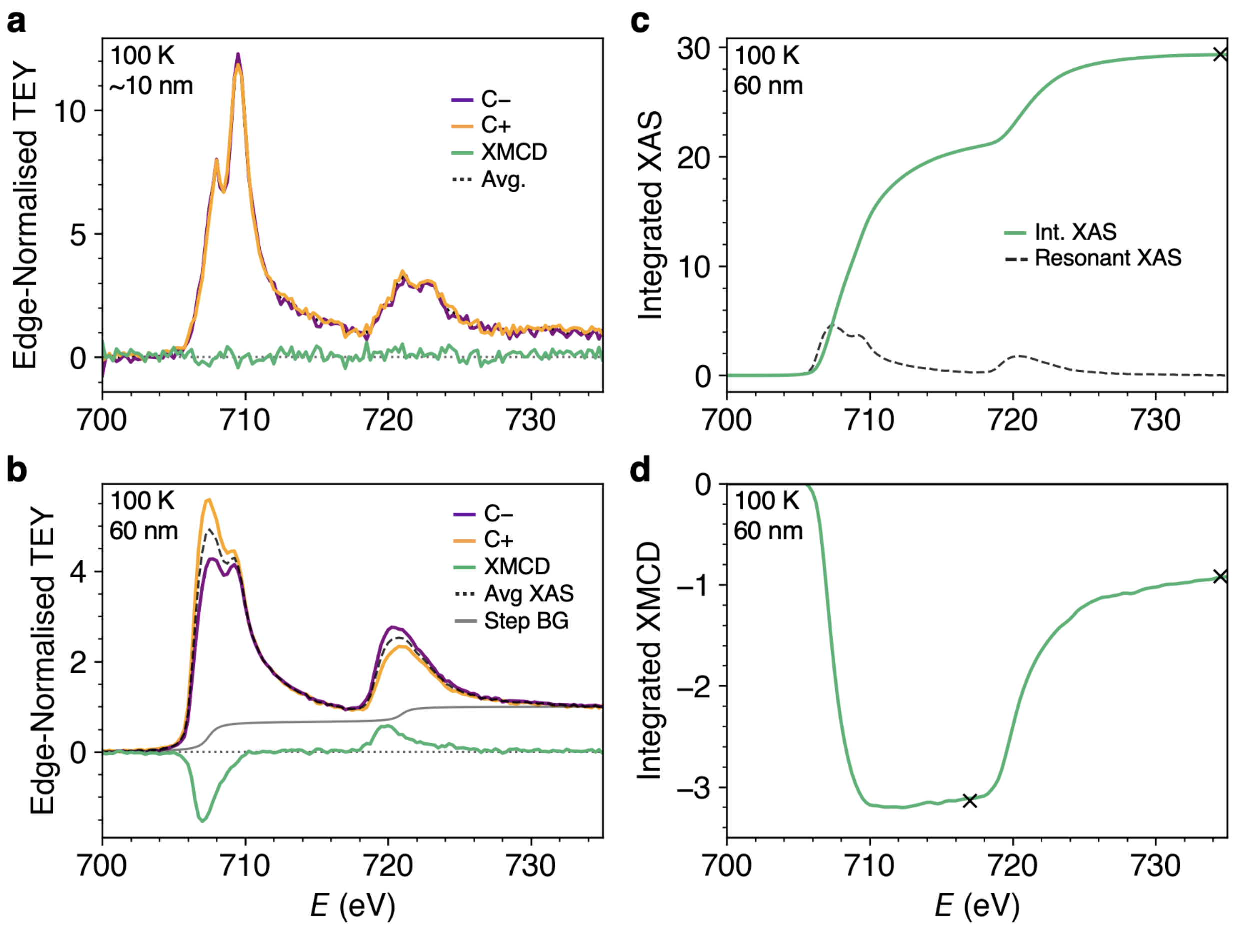

Figure S6 | **Flake sample x-ray absorption spectra measured in transmission. a-h**, X-ray absorption spectra (XAS) measured at the Fe L_3 and L_3 edges measured for the 15 and 60 nm thickness regions of the Fe_3GeTe_2 (FGT) flake sample. Spectra measured at ± 250 mT are plotted (purple and orange) alongside the calculated x-ray magnetic circular dichroism (XMCD) signal (green) for the 15 and 60 nm thickness regions at a range of temperatures. At 125K and below, the applied field was no longer sufficient to switch the magnetisation of the sample, so the circular polarisation (C±) was selected instead.

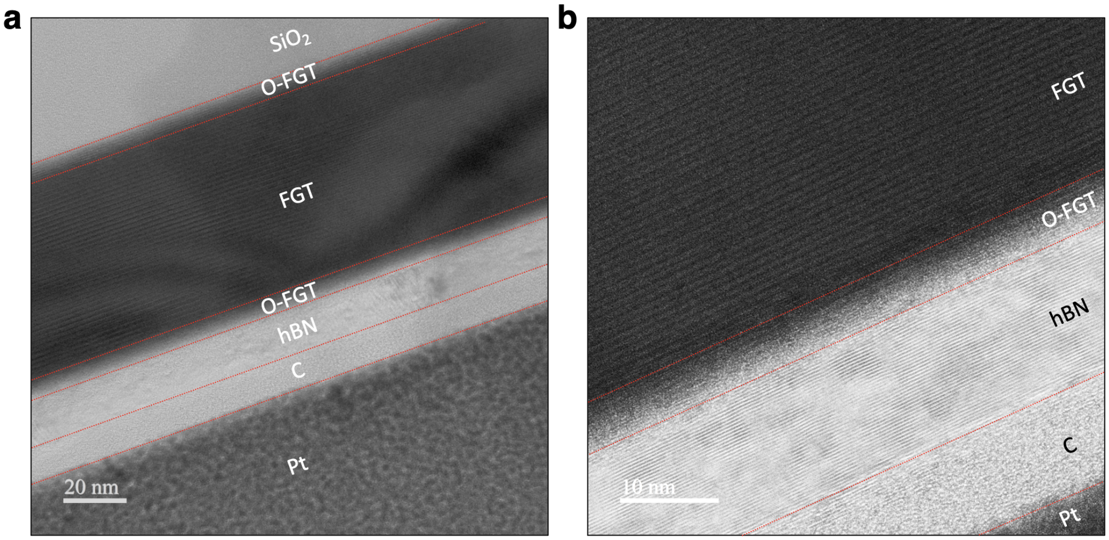

Figure S7 | **Oxide thickness determination. a**, **b**, Transmission electron microscopy images of a cross section of an Fe_3GeTe_2 (FGT) flake stamped onto a SiO_2 substrate. The images are presented with the square root of the intensity to improve the contrast of the lower intensity material layers within the FGT. The layers of stacked material are labelled: SiO_2, oxidised FGT (O-FGT), FGT, hexaboron nitride (hBN), C and Pt. The thickness of the oxidised FGT layer is approximately 7 nm.

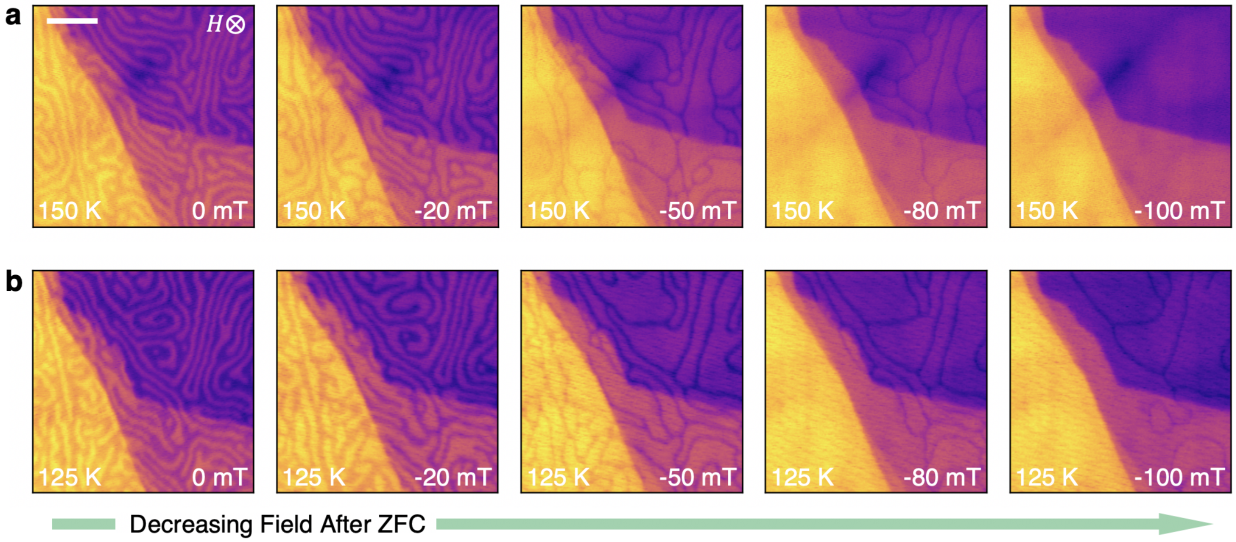

Figure S8 | **X-ray microscopy imaging results following the zero field-cooling procedure. a**,**b**, X-ray microscopy images measured following the zero-field cooling (ZFC) measurement procedure at 150 K and 125 K respectively. The green arrow indicates the measurement direction of the applied magnetic field. The scale bar is 1 μm.

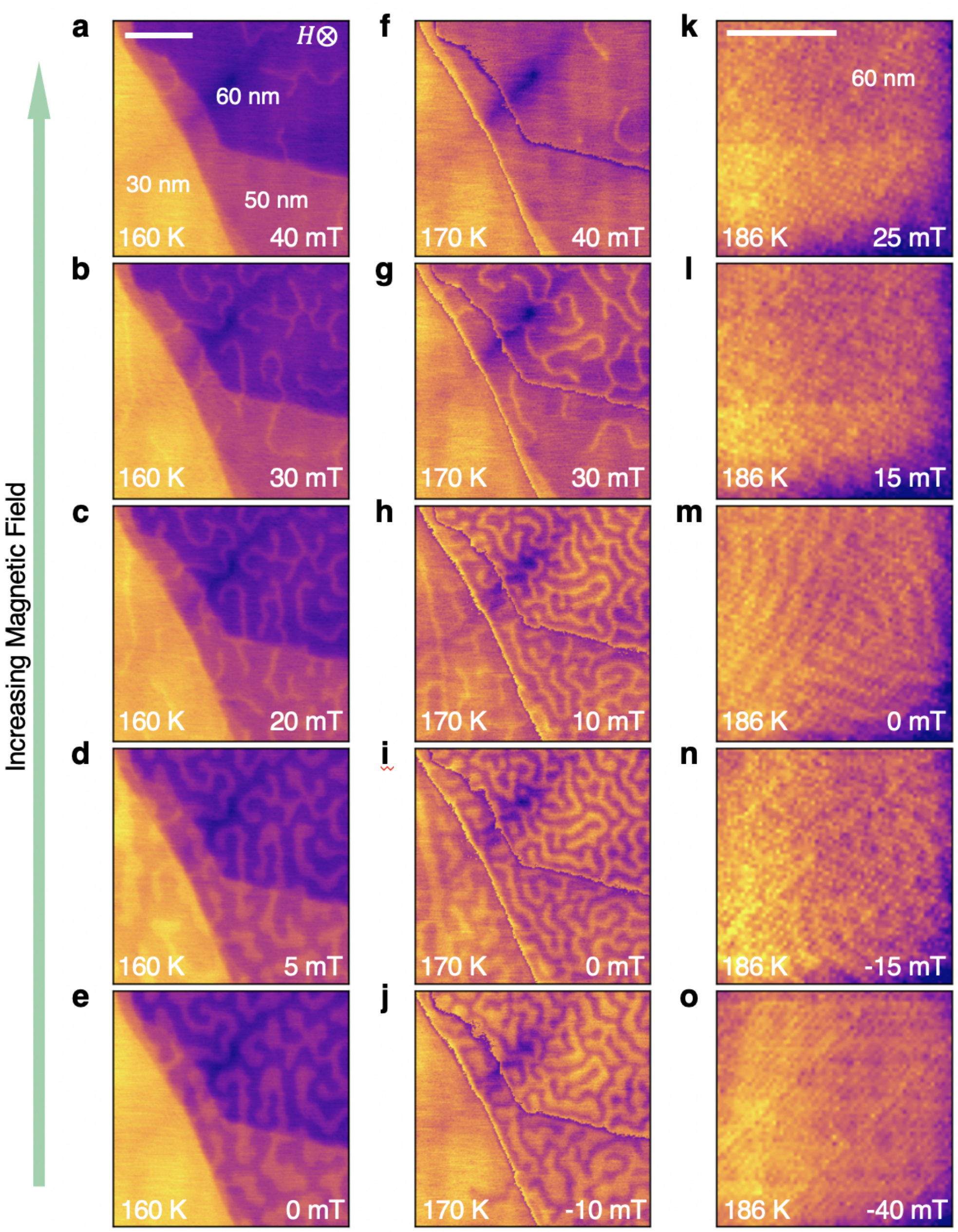

Figure S9 | **Additional data sets for the field sweep x-ray microscopy measurements. a-o**, X-ray microscopy images measured following the field sweep measurement procedure at 160 K, 170 K and 186 K. The green arrow indicates the measurement direction of the applied magnetic field. The scale bar is 1 μm.

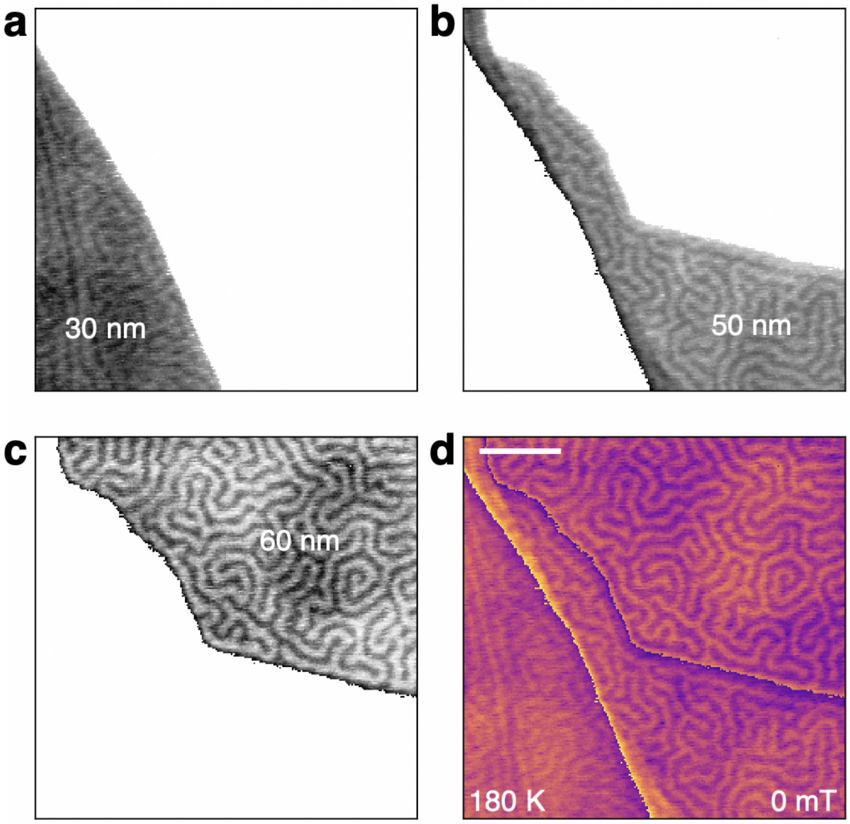

Figure S10 | **Example of contrast normalisation. a-c**, The isolated and masked regions of the x-ray microscopy image for each thickness region, 30, 50 and 60 nm respectively. **d**, The summation of the three isolated thickness regions, resulting in a contrast normalised x-ray microscopy image. The scale bar is 1 μm.

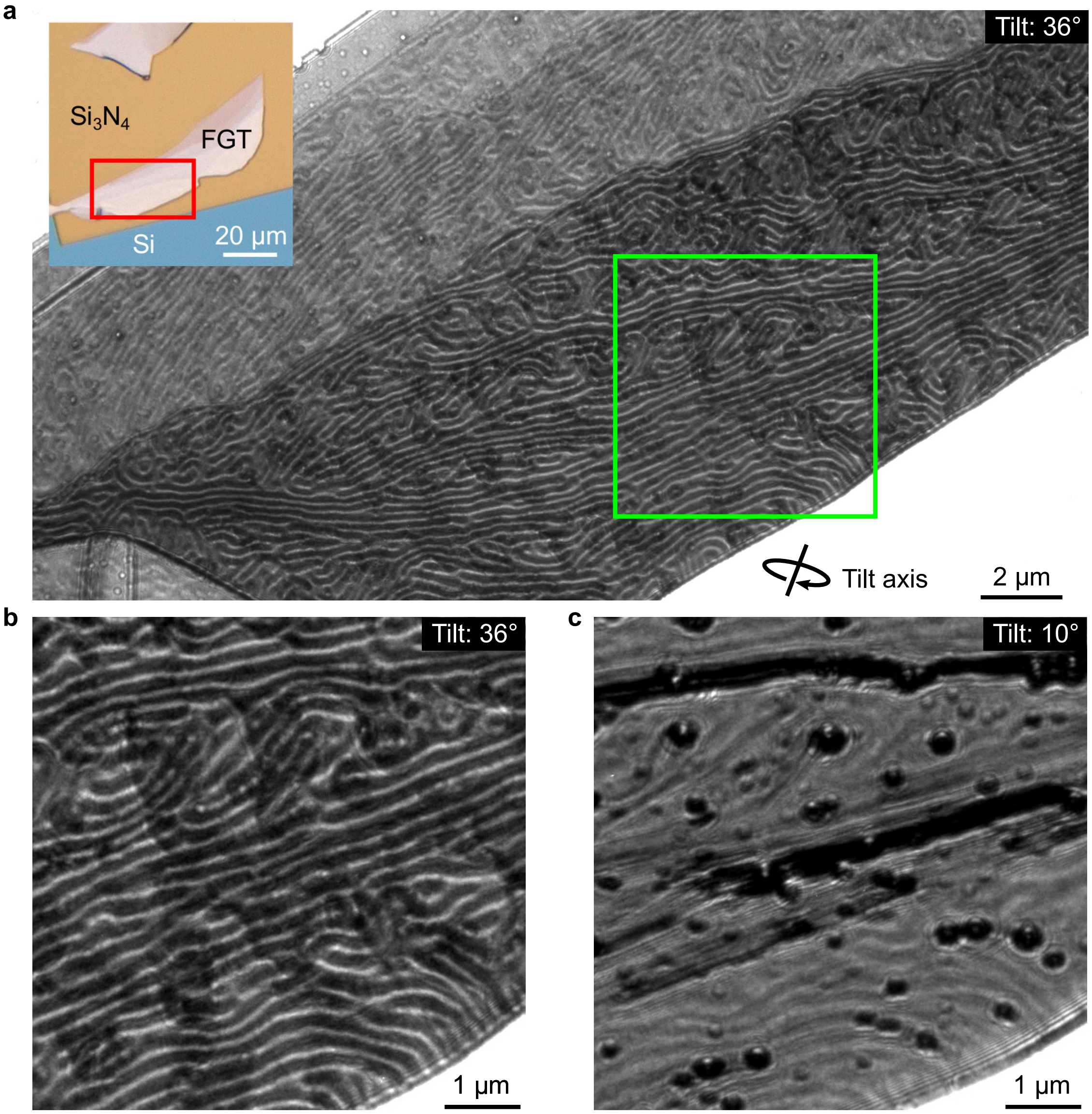

Figure S11 | **Lorentz transmission electron microscopy images of a tilted flake.** **a**, Lorentz transmission electron micrograph (LTEM) of an Fe_3GeTe_2 (FGT) flake acquired at 92 K in zero applied magnetic field at a defocus of 5.11 mm. Instead of lying normal to the electron beam, the sample was tilted to 36° about the axis shown. The inset shows an optical micrograph of the flake mounted on a Si_3N_4 membrane and the area from which the LTEM image was acquired is outlined in red. **b**, An enlargement of the image in **a** from the area outlined in green. **c**, An image from the same area as **b** acquired under the same conditions but the sample was tilted to 10° about the same axis.

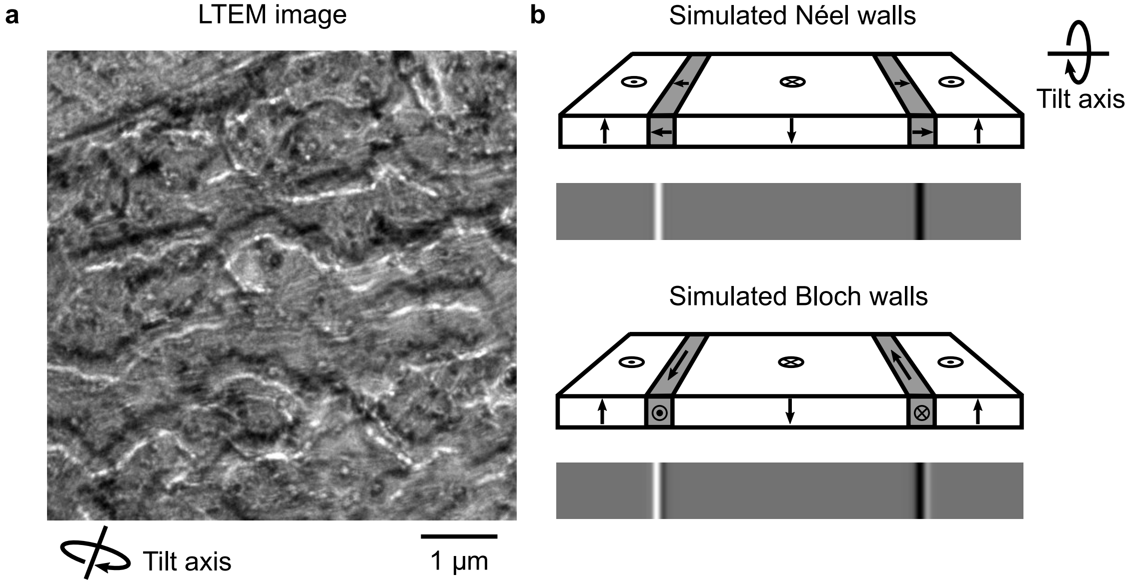

Figure S12 | **Distinguishing Néel and Bloch domain walls.** **a**, Lorentz transmission electron micrograph (LTEM) of the Fe_3GeTe_2 (FGT) flake acquired at 92 K with a defocus of 5.11 mm. No magnetic field was applied and the sample was tilted 36° about the axis shown. Magnetic domain walls appear as black and white lines. **b**, Visualisations and simulated images of Néel and Bloch magnetic domain walls under the same conditions as the imaged sample. The diagrams show the thin specimen in perspective view. The direction of the magnetization is indicated by arrows and the domain walls are shaded. The electron beam impinges from above but instead of lying normal to the beam, the plane of the specimen is tilted about the axis shown to match the experimental conditions. Simulated LTEM images for the two cases are shown beneath the diagrams.

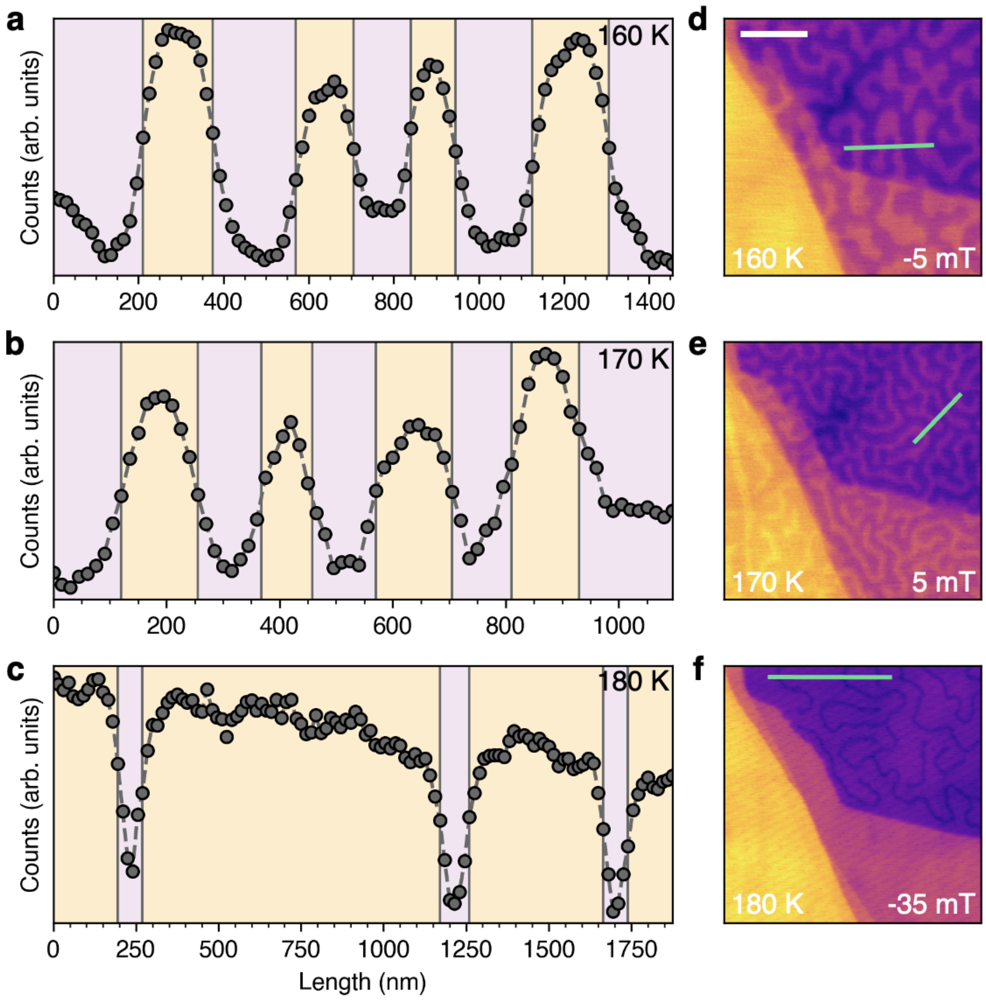

Figure S13 | **Example of the domain size determination.** **a-c**, Example line scans (left) utilised to the determine the average domain size at each temperature and applied magnetic field. The filled colour regions indicate the location of the determined domain boundaries. On the right, the original x-ray microscopy image is shown with the location of the line cut indicated by the green line. The scale bar is 1 μm.

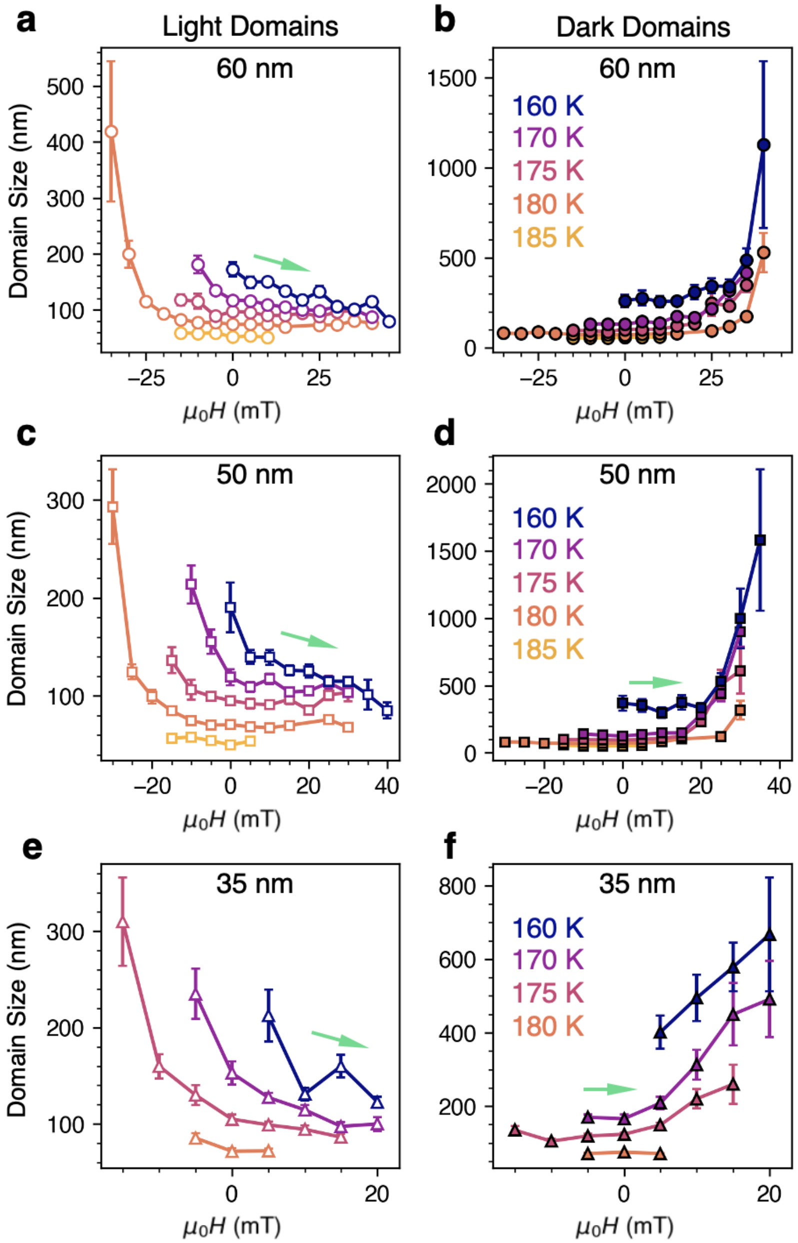

Figure S14 | **Extended data of all determined domain sizes.** **a-f**, The average domain size, for light and dark contrast domains, obtained from the x-ray microscopy images as a function of temperature T when following the field-sweep (FS) measurement procedure. Data is shown across a range of temperatures for the three thickness regions: 35, 50, and 60 nm. Error bars indicate the standard error determined from the sample of measured domain sizes.

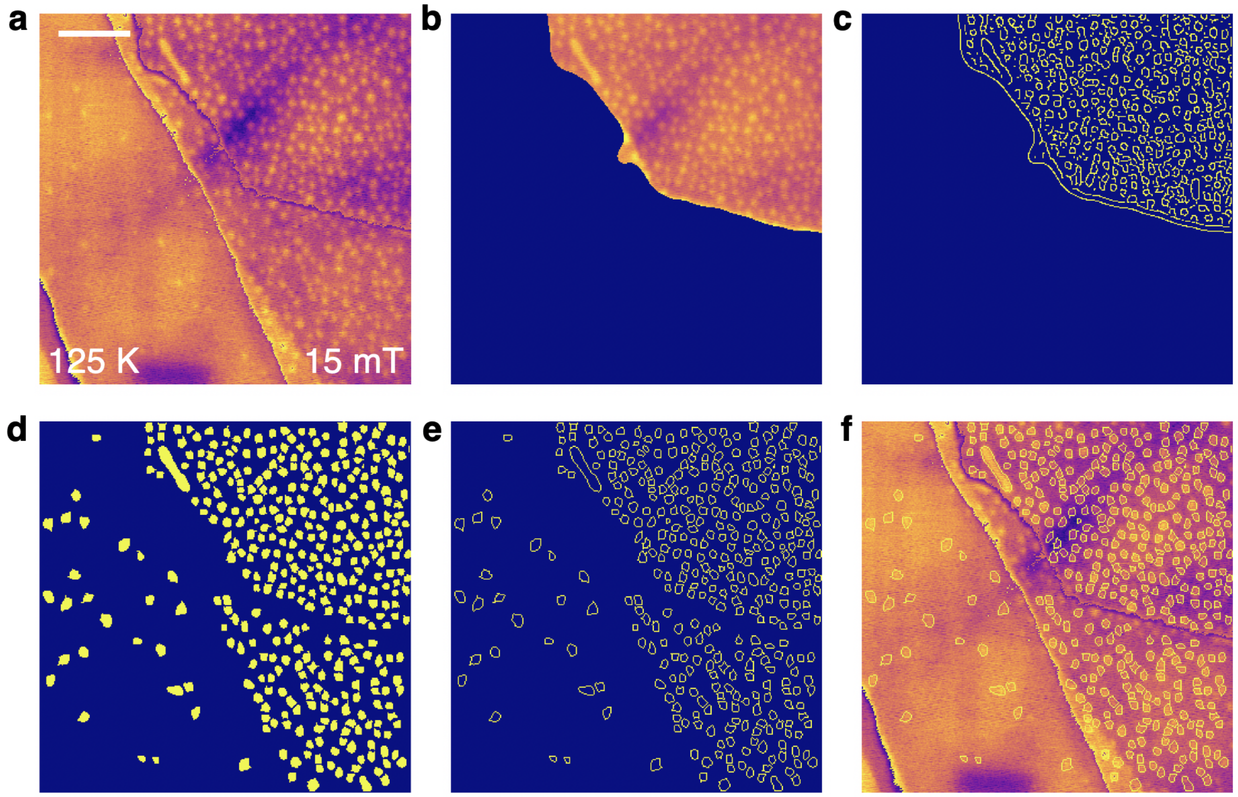

Figure S15 | **Example of the image recognition skyrmion fitting.** **a**, The original x-ray microscopy image, where each thickness region has been contrast normalised to highlight the magnetic texture. **b**, An example showing how each thickness region was masked. **c**, The initial results of the image recognition code detecting the skyrmions in the 60 nm region. **d**, The manually cleaned-up image detection data for all three thickness regions, showing the determined skyrmion bubble areas. **e**, The contours of the determined skyrmion bubbles. **f**, The contours of the determined skyrmion bubbles plotted over the contrast normalised x-ray microscopy image. The scale bar is 1 μm.

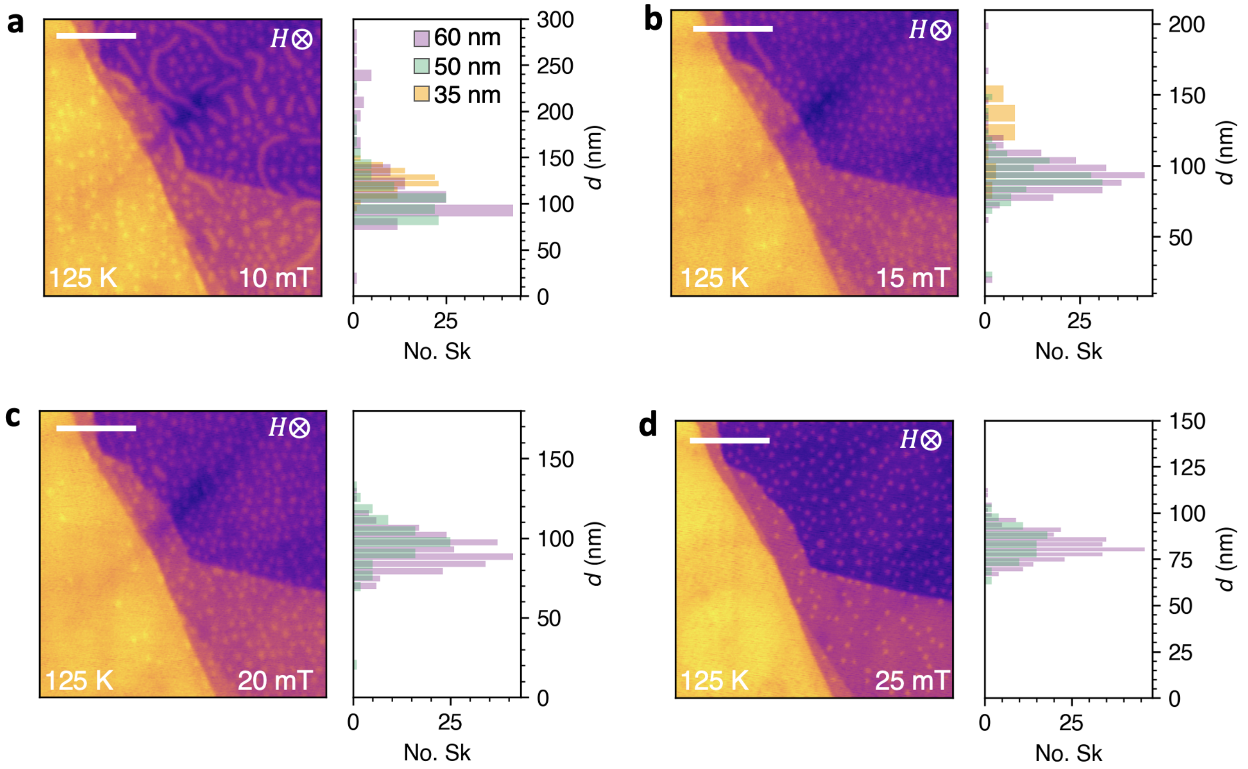

Figure S16 | **Formation of skyrmion after field-cooling. a-d**, X-ray microscopy images of the FGT flake acquired after field-cooling (FC) the sample from 200 K to 125 K at different applied magnetic fields. The histograms plot the distribution of the skyrmion size in the 60 nm (purple), 50 nm (green) and 30 nm (yellow) thickness regions, as determined by image recognition software. The scale bars are 1 μm.

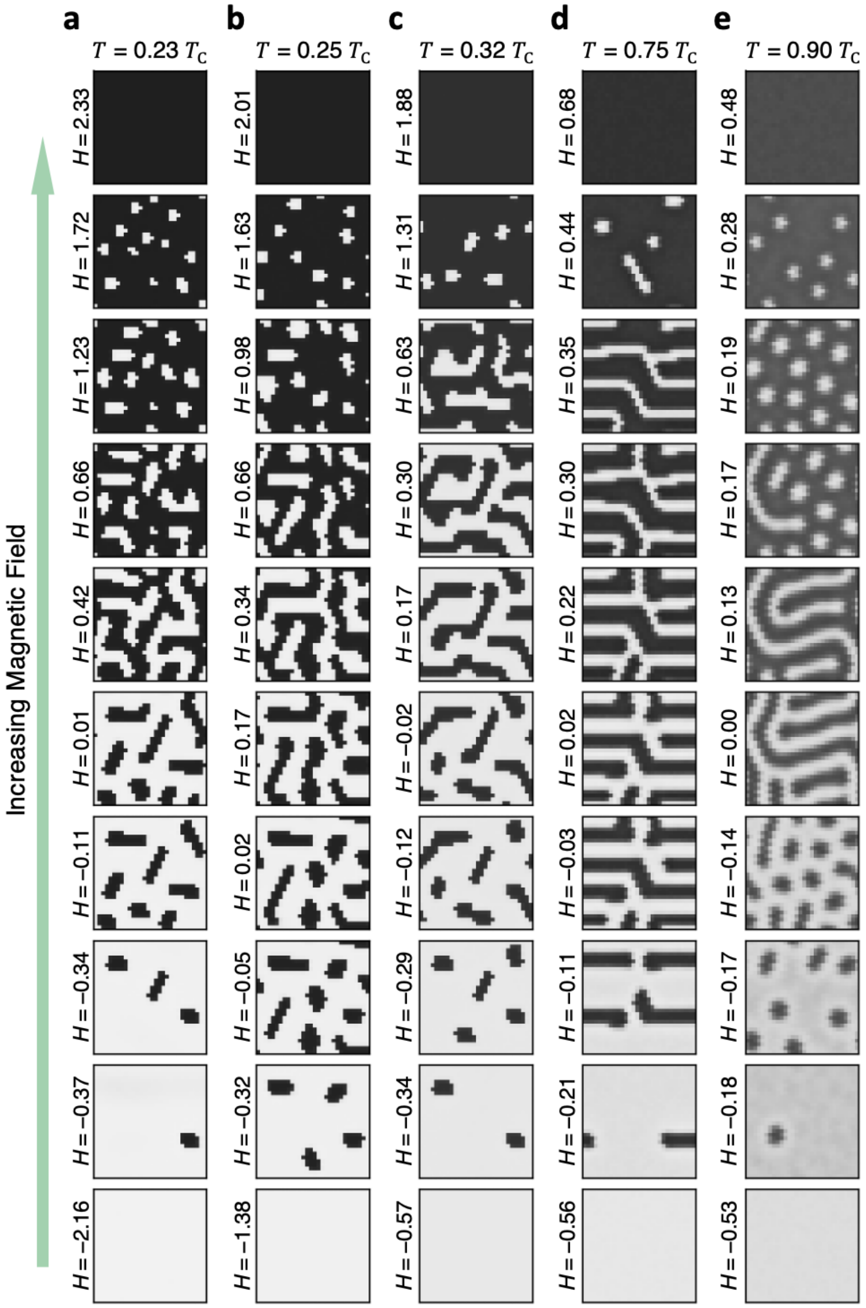

Figure S17 | **Mean-field simulations without modified anisotropy scaling. a-e**, Mean-field simulations of the magnetic domain structure evolution following the field sweep procedure for increasing applied field, H, at different temperatures, utilising the unmodified Callen-Callen anisotropy scaling model.

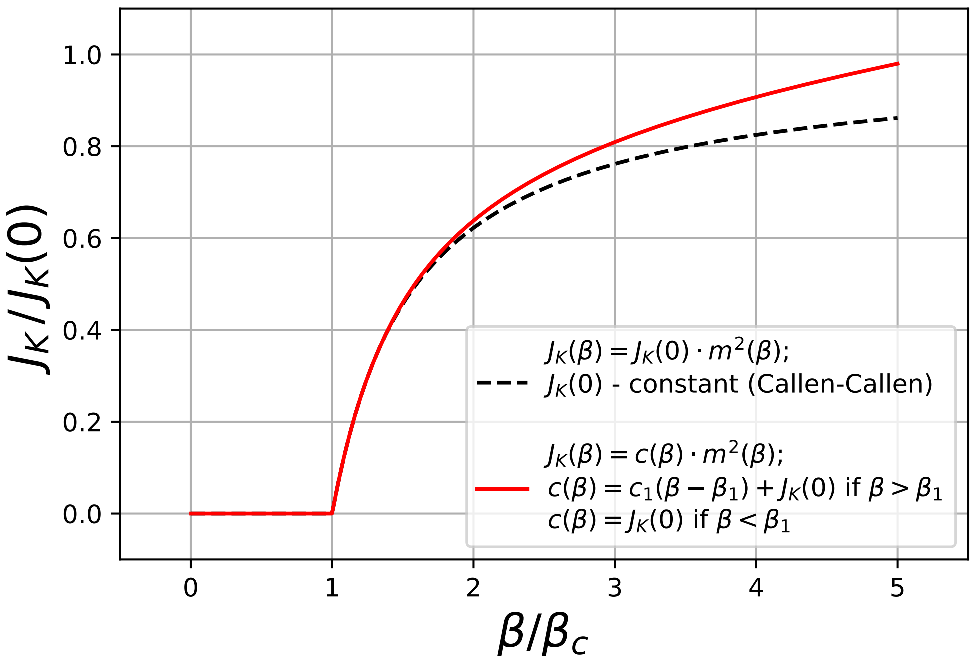

Figure S18 | **Temperature dependence of the mean-field effective anisotropy.** The effective anisotropy utilised in the mean-field simulations plotted as a function of $\beta = (k_\mathrm{B}T)^{-1}$. The dashed line represents the temperature dependence as given by the Callen-Callen relation defined by Eq. (3). The solid line represents the modified scaling to enhance the rate of anisotropy increase with decreasing temperatures (increasing β), defined by Eq. (4).
